# Distribution of Salivary Testosterone in Men and Women in a British General Population-Based Sample: The Third National Survey of Sexual Attitudes and Lifestyles (Natsal-3)

**DOI:** 10.1210/js.2016-1029

**Published:** 2017-01-12

**Authors:** Brian G. Keevil, Soazig Clifton, Clare Tanton, Wendy Macdowall, Andrew J. Copas, David Lee, Nigel Field, Kirstin R. Mitchell, Pam Sonnenberg, John Bancroft, Cath H. Mercer, Anne M. Johnson, Kaye Wellings, Frederick C. W. Wu

**Affiliations:** 1 Department of Clinical Biochemistry, University Hospital South Manchester, Manchester Academic Health Science Centre,; 2 Cathie Marsh Institute for Social Research, School of Social Sciences, and; 3 Andrology Research Unit, Manchester Centre of Endocrinology and Diabetes, Manchester Academic Health Science Centre, The University of Manchester, Manchester M13 9PL, United Kingdom;; 4 Research Department of Infection and Population Health, University College London, London WC1E 6BT, United Kingdom;; 5 Department of Social and Environmental Health Research, London School of Hygiene and Tropical Medicine, London WC1E 7HT, United Kingdom;; 6 Medical Research Council/Chief Scientist Office Social and Public Health Sciences Unit, University of Glasgow, Glasgow G4 0SF, United Kingdom; and; 7 Kinsey Institute, Indiana University, Bloomington, Indiana 47405

**Keywords:** saliva, testosterone, liquid chromatography-tandem mass spectrometry, LC-MS, population

## Abstract

**Introduction:**

Measurement of salivary testosterone (Sal-T) to assess androgen status offers important potential advantages in epidemiological research. The utility of the method depends on the interpretation of the results against robustly determined population distributions, which are currently lacking.

**Aim:**

To determine age-specific Sal-T population distributions for men and women.

**Methods:**

Morning saliva samples were obtained from participants in the third National Survey of Sexual Attitudes and Lifestyles, a probability sample survey of the British general population. Sal-T was measured using liquid chromatography-tandem mass spectrometry (LC-MS/MS). Linear and quantile regression analyses were used to determine the age-specific 2.5th and 97.5th percentiles for the general population (1675 men and 2453 women) and the population with health exclusions (1145 men and 1276 women).

**Results:**

In the general population, the mean Sal-T level in men decreased from 322.6 pmol/L at 18 years of age to 153.9 pmol/L at 69 years of age. In women, the decrease in the geometric mean Sal-T level was from 39.8 pmol/L at 18 years of age to 19.5 pmol/L at 74 years of age. The annual decrease varied with age, with an average of 1.0% to 1.4% in men and 1.3% to 1.5% in women. For women, the 2.5th percentile fell below the detection limit (<6.5 pmol/L) from age 52 years onward. The mean Sal-T level was approximately 6 times greater in men than in women, and this remained constant over the age range. The Sal-T level was lowest for men and highest for women in the summer. The results were similar for the general population with exclusions.

**Conclusions:**

To our knowledge, this is the first study to describe the sex- and age-specific distributions for Sal-T in a large representative population using a specific and sensitive LC-MS/MS technique. The present data can inform future population research by facilitating the interpretation of Sal-T results as a marker of androgen status.

## Teaser

Précis: We studied the population distribution of salivary testosterone (Sal-T) levels using liquid chromatography-tandem mass spectrometry and found that the mean Sal-T level was approximately 6 times greater in men than in women and decreased with age in both sexes.

The use of saliva in the investigation of testosterone status is attractive because sample collection is convenient, requires minimal training, and can be easily undertaken at home. Measurement of salivary testosterone (Sal-T), therefore, offers great potential in facilitating epidemiological and biomedical research at the population level.

Most testosterone circulating in the blood is bound to sex hormone binding globulin (SHBG) and albumin, rendering only a small free (unbound) fraction (~1% to 2%) [1] able to diffuse across capillaries into target tissues, where it exerts biological activity. Direct measurement of serum free testosterone (the reference standard) is technically challenging and expensive; hence, serum free testosterone is usually derived from mathematical formulas using association constants of testosterone with its binding proteins [2]. However, the relationship of calculated serum free testosterone to directly measured free testosterone and the clinical significance have not been universally accepted [3]. Testosterone circulating in the body readily diffuses across capillaries and salivary ducts, resulting in a salivary fraction containing free unbound testosterone [4]. Measurement of Sal-T might thus provide an alternative to measuring serum total testosterone, free testosterone, or bioavailable testosterone in the assessment of androgen status. Concerns have been raised regarding the reliability of Sal-T measurement using immunoassay methods [5]. However, recent methodological advances have allowed Sal-T to be reliably and accurately measured using more specific and sensitive liquid chromatography tandem mass spectrometry (LC-MS/MS) [6–8]. In both men and women, Sal-T correlates well with the calculated serum free testosterone level [8] and does not correlate with SHBG [9]. A high correlation between Sal-T and serum free testosterone measured by equilibrium dialysis in both men and women has also been confirmed; however, a substantial systematic positive bias was present in women, which might reflect the influence of salivary protein binding to the lower female concentrations of Sal-T [8]. Whether Sal-T can be a surrogate for circulating free testosterone or a valid measure of tissue bioavailable testosterone can now be investigated further.

Application of Sal-T measurements for the assessment of androgen status in men and women is critically dependent on the interpretation of results against rigorously determined age-specific population distributions. Using relatively small numbers of samples from hospital personnel or clinic attenders for this purpose is convenient but problematic owing to inherent selection bias and inadequate statistical power. Population ranges based on probability samples are more representative of the general population. They have only become available recently for serum testosterone measurements in both men [10] and women [11] and, as yet, have not been widely used. The present study aimed to determine the age-specific population distributions for Sal-T in a large sample of adult men and women from the general population in Britain using a highly sensitive and specific LC-MS/MS method. We have provided population distributions for the general population with exclusions (excluding those with self-reported medical conditions or using medications that can alter the testosterone levels) and the general population across the full age range to maximize its usefulness for a broad range of research studies.

## 1. Methods

### A. Study Population

The third National Survey of Sexual Attitudes and Lifestyles (Natsal-3) is a stratified probability sample survey of 15,162 men and women aged 16 to 74 years and resident in Britain, which used the postcode address file as its sampling frame. Participants were interviewed between September 2010 and August 2012 using computer-assisted personal interviewing, including a computer-assisted self-interview for the more sensitive questions. The response rate was 57.7%. Full details of the methods used have been described previously [12, 13].

After the interview, a subsample of men and women aged 18 to 74 years, who did not regularly work night shifts, were invited to provide a saliva sample to test for testosterone, without a return of the results. Consenting participants were given a self-collection pack and asked to provide their sample before 10 am to minimize the diurnal variation in testosterone [7]. They were asked not to brush their teeth, eat, or chew before giving the sample and to spit directly into a plain polystyrene tube. The saliva samples were posted to the laboratory, where they were prepared and frozen at −80°C until analysis [7]. On receipt of the sample, the participants were sent a £5 voucher as a token of appreciation.

Of 13,431 participants aged 18 to 74 years who did not regularly work at night, 9170 were invited to provide a saliva sample. A total of 4591 samples were received and matched to the survey data (50.1% of those invited to provide a sample). Of the samples, 463 (10.1%) were excluded (insufficient volume, n = 154; sample discolored or bloody, n = 91; sample recorded as taken after 10:30 am, n = 34; >5 days between the sample being taken and received by the laboratory or interval unknown because date of collection missing, n = 172; and not tested because of error, n = 12), leaving 4128 samples (45.0%) with a testosterone result.

The analysis for the general population included all 4128 participants (1675 men and 2453 women) with usable testosterone results. To generate the distribution for a general population with exclusions (those who did not report health conditions or taking medication that can influence testosterone levels), 530 men and 1177 women were excluded from analysis (individuals could be excluded for >1 reason). The reasons included prostate cancer (13 men), prostate enlargement (90 men), prostate surgery (20 men), and polycystic ovaries (35 women). The exclusions also included treatment of any of the following in the previous year: cancer, 25 men and 49 women; thyroid conditions, 27 men and 183 women; testicular or pituitary conditions, 16 men; and ovarian or pituitary conditions, 23 women. Also, we excluded participants if they were taking prescription medication for epilepsy (15 men and 15 women), had undergone hysterectomy and were taking hormone replacement therapy (to indicate oophorectomy; 181 women), and because of unprompted reported use of testosterone (1 man). We did not ask participants directly regarding their use of testosterone. Additionally, we excluded 363 men with a body mass index (BMI) <18.5 or >30 kg/m^2^ and 118 women with a BMI <18.5 or >40 kg/m^2^. Women reporting the current use of either hormonal replacement therapy (62 women) or hormonal contraception (pill, intrauterine device, injections, implants, or patch; 535 women) and those who were currently pregnant (42 women) were also excluded. Finally, those who did not answer any of the above questions were excluded (42 men and 134 women), leaving 1145 men and 1276 women for the analysis of the general population with exclusions.

### B. Measurements

The LC-MS/MS Sal-T assay was developed using strict validation criteria [7, 14]. Sample preparation using liquid–liquid extraction entailed adding sample (200 μL), D_5_-testosterone internal standard (10 μL; 340 pmol/L), and methyl-tert-butyl ether (1 mL). After vortexing for 5 minutes, the organic layer was transferred and evaporated and the residue reconstituted with a 500-mL/L methanol mobile phase (80 μL) and transferred to a 96-well microtiter plate.

Liquid chromatography was performed with an ACQUITY Ultra Performance Liquid Chromatography system coupled to a Xevo TQ-S mass spectrometer (Waters Corporation, Manchester, UK) operated in positive ionization mode. The lower limit of quantification was 6.5 pmol/L, and the assay was linear to ≥52,000 pmol/L. The interassay coefficient of variation ± standard deviation (SD) and bias was 12.9% ± 1.7% and 1.2%; 9.8% ± 2.5% and 0.4%; and 4.5% ± 12.0% and 1.9% at a concentration of 12.9, 26.0, and 260 pmol/L, respectively. The intra-assay coefficient of variation ± SD and bias was 9.5% ± 1.3% and 0.8%; 5.5% ± 1.6% and 12.6%; and 2.1% ± 6.2% and 11.1% at a concentration of 12.9, 26.0, and 260 pmol/L, respectively. Recovery was 104% (range, 98.3% to 108.9%) [7].

### C. Statistical Analysis

Statistical analyses were performed using STATA, version 13.1 (StataCorp, College Station, TX), accounting for the complex survey design (stratification, clustering, and weighting of the sample). We applied 2 weights when analyzing the data. The survey weight corrected for unequal probability of selection and differential response (by age, sex, and region) to the survey itself, and the saliva weight corrected for unequal probability of selection and differential response to the saliva sample. A number of factors were associated with providing a sample, including age at interview, ethnicity, general health, and sexual function measured using the Natsal sexual function questionnaire [15]. The full details of these weights and their calculation have been previously reported [12].

We used 2 statistical approaches to estimate the 2.5th to 97.5th percentiles for the population distributions for Sal-T levels in men and women: linear regression and quantile regression, as previously reported for calculating the serum testosterone reference ranges [10, 11]. Both analyses were performed to produce the distribution limits for the general population and the general population with exclusions.

Linear regression, as a parametric technique, can be unduly affected by extreme values. Therefore, very high Sal-T values were censored such that for each 10-year age group stratified by sex, values greater than the 99th percentile were replaced by the 99th percentile (17 men and 26 women). The 99th percentile values ranged from 587.4 pmol/L in the youngest men to 352.6 pmol/L in the oldest men and from 233.2 pmol/L in the youngest women to 104.6 pmol/L in the oldest women, respectively. The Sal-T data for men were approximately normally distributed; however, the distribution for women was skewed. Thus, the values were transformed on the natural log scale for analysis and back-transformed to generate the final population distribution limits. Because quantile regression is a nonparametric approach, it was not necessary to censor the extreme high values of Sal-T or to transform the data for the women.

Three men (all aged >60 years, all included in the general population with exclusions) and 76 women (distributed across the 18 to 74-year age range, 33 of whom were included in the general population with exclusions) had Sal-T levels less than the limit of detection (<6.5 pmol/L). Interval regression was used, assigning these cases to the range of 0 to 6.5 pmol/L for the linear regression for men. The lower bound for the women was set as 0.5 to allow the values to be log transformed. For quantile regression, these cases were assigned a value of 3.25 pmol/L (one-half the limit of detection).

For both men and women, the SD of Sal-T was not constant with age. Therefore, after fitting the linear regression for the mean values, we calculated the SD of the Sal-T levels for each year of age and used these values as the outcome in a second linear regression analysis to predict the SD as a function of age. The predicted 2.5th and 97.5th percentiles for each year of age were calculated as the predicted mean Sal-T minus the predicted SD for that age multiplied by the lower bound (b_l_) and the predicted mean plus the predicted SD multiplied by the upper bound (b_u_), respectively, with b_l_ and b_u_ selected such that across all ages, 2.5% of the population had testosterone values less than the lower bounds and 2.5% of the population had testosterone values greater than the upper bounds. We tried different values for each multiplier, b_l_ and b_u_, starting with 1.96, which corresponded to the normal distribution, and iteratively increasing or decreasing the values until we achieved the desired coverage. For men in the general population, the values were b_l_ of 2.00 and b_u_ of 2.30, and for women in the general population, they were b_l_ of 2.11 and b_u_ of 1.96. The values for the men in the general population with exclusions were b_l_ of 2.09 and b_u_ of 2.25, and for women, b_l_ of 2.10 and b_u_ of 1.96.

For men, the SD of Sal-T decreased with age up to a point (from approximately age 70 years) and increased again in the oldest age group. We were unable to adequately model this increase in the SD to accurately calculate the 2.5th and 97.5th percentiles (which are based on the SD) and consequently truncated the population distribution analysis for men at age 69. No equivalent increase in the SD was found among older women; therefore, the data are presented for the full age range, 18 to 74 years. Truncation was not necessary for the analysis of the mean testosterone levels, including associations with seasonal changes.

To allow for a possible nonlinear relationship between Sal-T and age, we explored 2 different functions of age (in addition to a linear function) in both the linear and the quantile regression analyses: a quadratic function and a restricted cubic spline function. For the latter, 3 knots were specified at the 10th, 50th, and 90th percentiles of age (the default placement for 3 knots). The population distribution produced by the models using the quadratic and cubic spline functions was similar; therefore, we opted to use the simpler quadratic function in the final models. For women, the analyses were performed on log-transformed data and the data were back-transformed; therefore, the geometric mean values are presented.

To assess the seasonal variation in testosterone, the mean (geometric mean for women) testosterone and 95% confidence intervals were plotted by season for the general population, and linear regression was used to test for differences. Each season was defined as winter (December, January, and February), spring (March, April, and May), summer (June, July, and August), and autumn (September, October, and November). To explore potential geographical differences, the participants were grouped into 3 broad regions of residence: Scotland and North of England, Midlands and Wales, and East and South of England (including London).

### D. Ethics Statement

The Oxfordshire Research Ethics Committee A approved Natsal-3 (reference no. 09/H0604/27). All participants provided written informed consent for anonymized testing of the saliva samples, without a return of the results.

## 2. Results

Distributions of the mean ± SD and median and interquartile range of the Sal-T levels in the general population by 10-year age group are listed in Table 1 and for the general population with exclusions in Supplemental Table 1. The Sal-T levels for both men and women showed a distinct age-related decline, with a clear demarcation in the mean levels between men and women. The mean Sal-T concentration was approximately 6 times greater in the men than in the women; this relationship remained constant over the 6 decades studied in both the general population and the general population with exclusions (Table 1; Supplemental Table 1).

**Table 1.** **Mean and Median Salivary Testosterone by Age Group and Sex in General Population**

Variable	Sal-T (pmol/L)		Denominator	
	Mean ± SD	Median (IQR)	UWt	Wt
Men				
18–24	314.8 ± 111.6	314.9 (246.3–384.1)	187	244
25–34	266.7 ± 102.5	264.6 (198.6–325.9)	249	335
35–44	232.6 ± 91.5	229 (178.4–285.3)	244	376
45–54	207.5 ± 80.2	203.2 (155.3–248.9)	305	397
55–64	174.4 ± 64.7	175.9 (130.6–214.9)	347	350
65–69	157.6 ± 58.5	152.0 (119.3–190.1)	194	153
Women				
18–24	51.1 ± 45.1	39.2 (21.7–65.6)	247	268
25–34	42.6 ± 32	37.1 (24.3–49.6)	441	403
35–44	41.1 ± 31.7	32.4 (21–50.7)	425	414
45–54	33.9 ± 28.5	26.6 (17.9–40.5)	451	430
55–64	27.6 ± 18.6	22.9 (15.3–35.8)	462	368
65–74	27.5 ± 20.2	23.2 (14.8–33.2)	427	284

Abbreviations: IQR, interquartile range (25 to 75th percentiles); UWt, unweighted; Wt, weighted.

**Figure 1.**
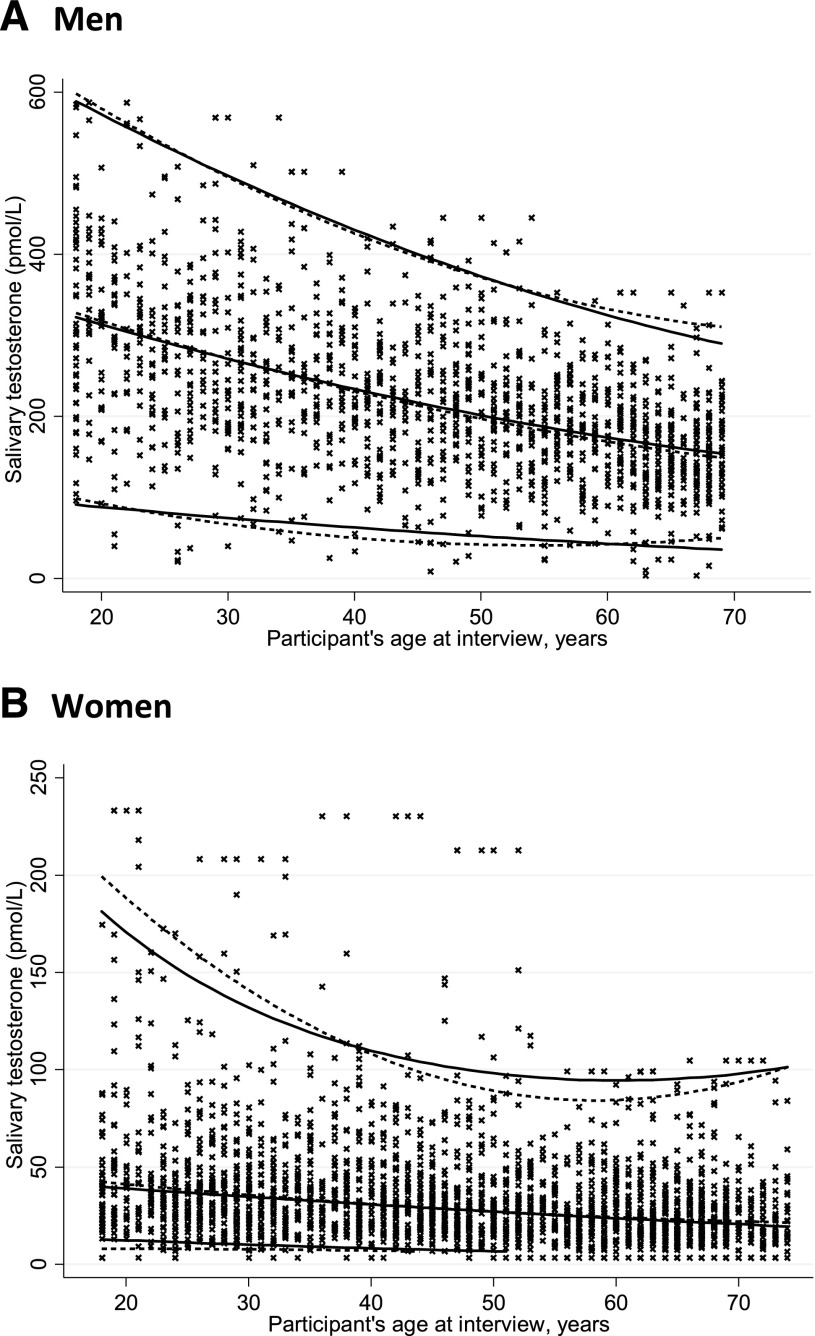
Distribution of salivary testosterone in (A) men and (B) women in the general population. Curves created using linear regression (solid line) for the fitted mean (men) or geometric mean (women) for the 2.5th and 97.5th percentiles and quantile regression (dashed line) for the median, 2.5th percentile, and 97.5th percentile. Observed values (*x*) for 1526 men and 2543 women displayed.

The Sal-T distributions according to the linear and quantile regression analyses for men and women in the general population are shown in Fig. 1. For both men and women, the linear and quantile regression analyses produced similar population distributions. Supplemental Table 2 shows the age-specific values for the 2.5th and 97.5th percentiles of the distribution for the general population produced by the linear regression (those produced by quantile regression analysis not shown). The Sal-T distributions for men and women in the general population with exclusions are shown in Supplemental Fig. 1, and the values for the 2.5th and 97.5th percentiles of the distribution are listed in Supplemental Table 2. For women, the 2.5th percentile fell below the limit of detection (<6.5 pmol/L) from age 52 years onward in the general population and age 54 years onward in the general population with exclusions; thus, these data are not provided.

The range of Sal-T values greater than the 97.5th percentile among women aged <55 years was wide; however, for those aged >55 years, most of the high values were clustered just above the 97.5th percentile line (Fig. 1). Although detailed information on menstrual phase was not collected, our questionnaire enabled the identification of women who had provided saliva samples within 7 days of starting their last menstrual period (presumed early follicular phase). Very few of the high values in the premenopausal women were among those in the early follicular phase (data not shown), suggesting that they might ay reflect mid-cycle testosterone peaks [16].

For the full age range examined, the mean Sal-T levels decreased by approximately 50% to 60% in both the general population with exclusions and the general population of men and women. Because our models of the association between Sal-T and age included a nonlinear function of age, the predicted year-by-year decline in testosterone varied by age. For men in the general population, the predicted decrease in the average Sal-T level for each year of age was 1.3% to 1.5%. The predicted decline between age 18 and 19 was 1.4% (range, 322.6 to 318.0 pmol/L), between age 45 and 46 was 1.5% (range, 216.9 to 212.7 pmol/L), and between age 68 and 69 was 1.3% (range, 156.0 to 153.9 pmol/L). For women in the general population, the predicted decrease in the average Sal-T level for each year of age was 1.0% to 1.4%. The decline between age 18 and 19 was 1.0% (range, 39.8 to 39.4 pmol/L), between age 45 and 46 was 1.4% (range, 28.9 to 28.5 pmol/L), and between age 73 and 74 was 1.0% (range, 19.7 to 19.5 pmol/L).

**Figure 2.**
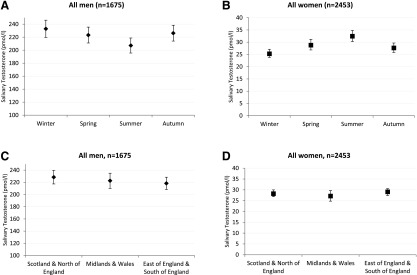
Mean (men) and geometric mean (women) salivary testosterone (pmol/L) by season [(A), men; (B) women] and region [(C) men; (D) women] in the general population.

Seasonal differences in the mean Sal-T levels were observed (*P* < 0.0001 for both men and women; Fig. 2); however, these differed by sex, with the lowest levels in the summer for men and the highest levels in the summer for women. We found no associations between the mean Sal-T level and the broad geographical region (*P* = 0.2432; Fig. 2).

## 3. Discussion

To our knowledge, the present study is the first to establish age-specific population distributions for LC-MS–analyzed Sal-T in men and women from a large general population sample. Our findings showed only a minor overlap between the age-specific male and female population distributions, mirroring those seen with serum testosterone, and lending support to the validity of our Sal-T measurements. The finding of sixfold greater Sal-T levels in the men compared with the women was also similar to that observed for serum testosterone [17], reflecting the markedly greater daily testosterone blood production rate in men [18]. A distinct age trend in Sal-T levels was observed in both sexes. The rate of cross-sectional decline in Sal-T with age was similar to the decline in Sal-T with age in other smaller studies of men [19–21] and women [20] but greater than the reported decline in serum testosterone in men [22–24] and women [11, 25, 26].

The age-associated decline in serum testosterone has been implicated in a variety of physiological changes in aging men [27, 28]. However, this has been disputed by some [29, 30], who have suggested that the apparent decline is largely due to comorbidity, with healthy elderly men showing little change in their circulating testosterone levels. Although we found that men aged >45 years who had not reported any of the exclusion health conditions had slightly greater levels of Sal-T compared with the whole sample, the Sal-T levels in these men had nevertheless decreased by one-half from age 18 to 69 years. This suggests that the widely reported serum total and free testosterone decreases during the life course of 17% and 35%, respectively [22–24], might underestimate the aging-associated decline in testicular function or testosterone bioavailability at the tissue level. An important corollary of this compelling age trend in Sal-T is to reinforce the view that a *Z*-score approach, using age-specific population ranges, might be more appropriate and physiologically meaningful than the previously preferred testosterone score approach using comparisons with ranges derived from young (age <40 years) healthy men [10].

In premenopausal women, we observed some extreme high values of Sal-T, extending far above the 97.5th percentile, which possibly reflected the midmenstrual cycle peaks in testosterone [16, 26, 31]. We did not collect detailed information on the menstrual phase; thus, we were unable to control for variations in testosterone across the menstrual cycle in our analysis. In broad agreement with the serum testosterone levels from other large population-based studies [11, 25, 26], we found that the decline in Sal-T in women was steepest in the early reproductive years and subsequently flattened out in midlife. In agreement also with the serum testosterone findings from other studies [11, 26, 32], we did not observe a substantial effect of the menopausal transition on Sal-T levels. The percentage of change in serum testosterone previously found in healthy women aged 20 to 60 years was 30% [11]. In contrast, the percentage of change in Sal-T in our study was ∼60% for a similar age range. Thus, just as in men, the age-related decrease in Sal-T levels in women who did not report any of the exclusion health conditions was appreciably greater than that observed for serum testosterone. The principal sources of androgens in postmenopausal women are the adrenal gland and the ovary [33]. An increase in free testosterone could also arise from a relative decrease in SHBG compared with testosterone, a finding consistent with the trend of decreasing SHBG across the menopausal transition [34].

The seasonal variation in Sal-T observed in men showed the opposite trend to that seen in women, with an increase in the summer and a decrease in the winter in women. Previous studies examining seasonal variations in serum testosterone levels in men and women have yielded inconsistent results, with either no seasonal variation found [35] or with peak levels found in the winter [36] or the summer [37]. In the only Sal-T study, peak levels were found in October and December for the women and men, respectively [38]. Although statistically significant, the magnitude of the observed seasonal differences in Sal-T was relatively small (~20 pmol/L in men and ~8 pmol/L in women), and the variation might not be biologically or clinically important. Given these inconsistencies, we do not believe it would be appropriate to provide separate Sal-T population distributions stratified by season.

Natsal-3 is broadly representative of the British population, including in terms of ethnicity [13], but was not designed specifically to examine ethnic variations in testosterone. We found no association with the broad geographical regions, which is perhaps unsurprising given that Britain is a small country in terms of area and previous research into geographical variation has been on a global scale [39].

The strengths of the present study are the large general population sample size, the state-of-the-art LC-MS/MS measurement of Sal-T, and the rigorous statistical analysis techniques. To enable the fullest application in future investigations, we established population distributions, not only for the entire general population, but also after exclusion of conditions and medications that can affect Sal-T levels. This ensured applicability of the presented information to a wide range of epidemiological and biomedical studies in the future.

The present study also had some limitations. The health conditions were self-reported, and single morning saliva samples cannot account for intraindividual variations resulting from circhoral, diurnal, and circannual rhythms. The lack of accurate information on the timing of samples in relation to the menstrual cycle and clinical information on the presence of polycystic ovarian syndrome among women could have introduced added “noise” in the distributions. Although our sample was similar to the census with respect to ethnicity, health, and marital status after weighting [12, 13], just as with any general population survey, our data were susceptible to some participation biases. For instance, individuals in residential or nursing care were not included in the sampling frame, and poor health could have affected subjects’ willingness to participate (*i.e.,* our population distributions for the general population might refer to a slightly healthier sample than the true British general population). The final response rate to the saliva study was 45%; therefore, the saliva data were weighted during analysis to minimize the potential for a nonresponse bias [12].

Age- and sex-specific population distributions are important as a baseline against which other analyses and research studies can be compared. The array of background information, in particular, with respect to age and BMI, will be important when considering important research questions, such as the variations in Sal-T at the population level with respect to the frequency of sexual activity at the extremes of the age spectrum, sexual satisfaction, and number of sexual partners. Some of these questions are being addressed in our ongoing analyses.

The presented data describe the distribution of Sal-T in the general population as part of a large study to investigate the determinants of variations in sexual lifestyle and practices in men and women. The information is not intended to be applied to the clinical setting (without further stringent clinical evaluation), particularly with respect to hormone replacement therapy for older individuals. The very clear decline in Sal-T levels with age lends support to the view that lower testosterone levels are a physiological change and argue against the use of hormone replacement therapy for older individuals.

We have determined age-specific population distributions for Sal-T in a large, representative population of men and women using a highly specific and sensitive LC-MS/MS technique. The relative simplicity of saliva collection has important implications for large population-based studies, in which serum collection has been impractical or too expensive. These population data, which can be harmonized with those from other laboratories using validated LC-MS/MS methods, provide a benchmark for ensuring the appropriate interpretation and comparisons of Sal-T results in future research. An essential step has now been taken to allow the application of Sal-T levels in investigating the potential importance of androgen exposure in many aspects of sexual behavior and general health in largescale population surveys of men and women.
