# Supplementary material for: Distribution of Salivary Testosterone in Men and Women in a British General Population-Based Sample: The Third National Survey of Sexual Attitudes and Lifestyles (Natsal-3)
Source: J Endocr Soc. 2017 Jan 12;1(1):14–25. doi: 10.1210/js.2016-1029 (PMC5677216; doi:10.1210/js.2016-1029)
Supplement: Supplementary file 1 [file js-01-14-st1.docx]

| **Appendix table 1: Mean and median salivary testosterone (sal-T) by age group and gender in the general population with exclusions*** | | | | | | |
| --- | --- | --- | --- | --- | --- | --- |
|  |  | | | | | |
|  | sal-T (pmol/l) | | | | *denominator* | |
|  | mean | sd | median | (IQR) | *unwt* | *wt* |
| **Men** |  |  |  |  |  |  |
| 18-24 | 310.5 | 108.3 | 308.1 | (246.3-372.4) | *150* | *195* |
| 25-34 | 273.9 | 102.1 | 270.9 | (246.3-372.4) | *195* | *270* |
| 35-44 | 237.8 | 82.3 | 231 | (195.1-286.5) | *174* | *269* |
| 45-54 | 218.8 | 77.6 | 215.7 | (172.8-262.6) | *215* | *278* |
| 55-64 | 181.8 | 63.7 | 181.8 | (140.1-222.5) | *232* | *231* |
| 65-69 | 162.6 | 53.9 | 155.7 | (130.6-190.1) | *108* | *83* |
| All aged 18-69 | 237.5 | 96.9 | 228.2 | (173.8-293.8) | *1074* | *1326* |
| **Women** |  |  |  |  |  |  |
| 18-24 | 61.8 | 40.8 | 50.6 | (36.1-87.2) | *44* | *56* |
| 25-34 | 45.9 | 31.2 | 38.8 | (27.2-58.7) | *177* | *179* |
| 35-44 | 40.4 | 32 | 31.8 | (20.6-49.3) | *223* | *226* |
| 45-54 | 35.5 | 28.9 | 29.0 | (19.0-40.7) | *294* | *274* |
| 55-64 | 27.6 | 17.6 | 23.3 | (16.5-35.4) | *292* | *236* |
| 65-74 | 27.4 | 19 | 24.3 | (15.1-33.2) | *246* | *163* |
| All aged 18-74 | 36.6 | 28.9 | 29.6 | (19.2-44.2) | *1276* | *1134* |
| IQR=Interquartile range (25-75th percentiles) | | | |  |  |  |
| * Men with a body mass index (BMI) <18.5 or >30 kg/m^2^ and women with a BMI <18.5 or >40 kg/m^2^; prostate cancer, prostate enlargement, prostate surgery; polycystic ovaries; treatment for cancer, thyroid, testicular/ovarian or pituitary conditions in the past year; medication for epilepsy; women reporting both hysterectomy and HRT use; HRT; and hormonal contraception. All self-reported. See 'study population' section of methods for further details of exclusion criteria. | | | | | |  |
