# Supplementary material for: Distribution of Salivary Testosterone in Men and Women in a British General Population-Based Sample: The Third National Survey of Sexual Attitudes and Lifestyles (Natsal-3)
Source: J Endocr Soc. 2017 Jan 12;1(1):14–25. doi: 10.1210/js.2016-1029 (PMC5677216; doi:10.1210/js.2016-1029)
Supplement: Supplementary file 2 [file js-01-14-st2.docx]

| **Appendix table 2: Salivary testosterone 2.5th and 97.5th percentiles for men and women at selected years of age (linear regression)** | | | | |
| --- | --- | --- | --- | --- |
|  |  | |  | |
|  | **General population** | | **General population**  **with exclusions** | |
| Selected Age (years) | 2.5^th^ percentile | 97.5^th^ percentile | 2.5^th^ percentile | 97.5^th^ percentile |
| **Men** |  |  |  |  |
| 18 | 91 | 589 | 82 | 567 |
| 20 | 88 | 573 | 81 | 554 |
| 25 | 81 | 534 | 78 | 522 |
| 30 | 75 | 497 | 75 | 491 |
| 35 | 69 | 462 | 71 | 460 |
| 40 | 63 | 430 | 68 | 430 |
| 45 | 57 | 400 | 66 | 401 |
| 50 | 52 | 373 | 63 | 372 |
| 55 | 47 | 348 | 60 | 344 |
| 60 | 43 | 325 | 57 | 317 |
| 65 | 39 | 305 | 54 | 291 |
| 69 | 36 | 290 | 52 | 270 |
| **Women** |  |  |  |  |
| 18 | 13 | 181 | 18 | 144 |
| 20 | 12 | 171 | 16 | 139 |
| 25 | 11 | 149 | 14 | 129 |
| 30 | 10 | 132 | 12 | 119 |
| 35 | 9 | 119 | 10 | 112 |
| 40 | 8 | 110 | 9 | 106 |
| 45 | 7 | 103 | 8 | 101 |
| 50 | 7 | 101 | 7 | 97 |
| 55 | * | 98 | * | 93 |
| 60 | * | 98 | * | 91 |
| 65 | * | 96 | * | 90 |
| 70 | * | 95 | * | 89 |
| 74 | * | 96 | * | 89 |
|  |  |  |  |  |
| *<6.5pmol/l |  |  |  |  |
