# Supplementary material for: Distribution of Salivary Testosterone in Men and Women in a British General Population-Based Sample: The Third National Survey of Sexual Attitudes and Lifestyles (Natsal-3)
Source: J Endocr Soc. 2017 Jan 12;1(1):14–25. doi: 10.1210/js.2016-1029 (PMC5677216; doi:10.1210/js.2016-1029)
Supplement: Supplementary file 3 [file js-01-14-sf1.pptx]

## Slide 1
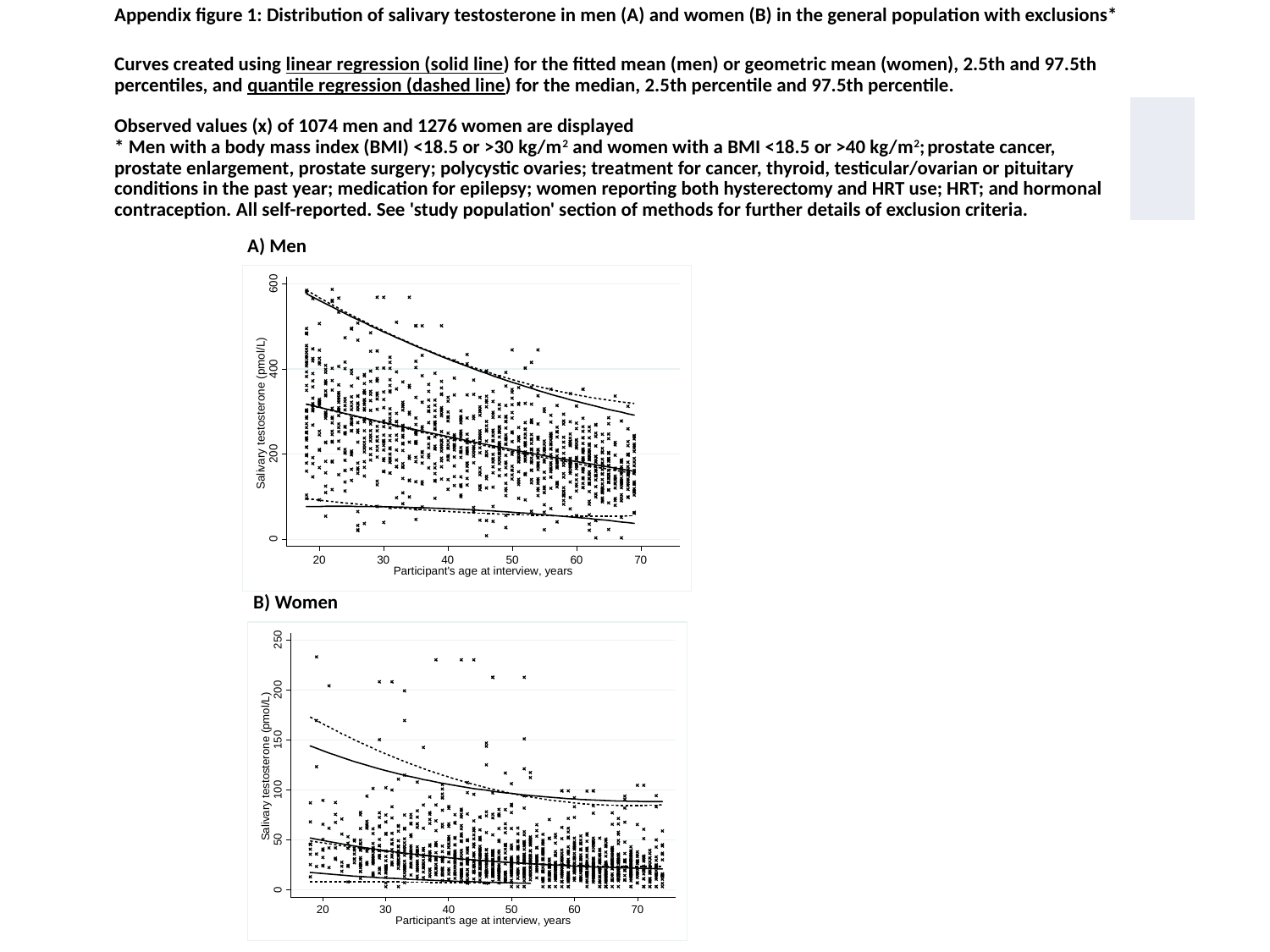

| Appendix figure 1: Distribution of salivary testosterone in men (A) and women (B) in the general population with exclusions\* | |
| --- | --- |
| Curves created using linear regression (solid line) for the fitted mean (men) or geometric mean (women), 2.5th and 97.5th percentiles, and quantile regression (dashed line) for the median, 2.5th percentile and 97.5th percentile. | |
| Observed values (x) of 1074 men and 1276 women are displayed \* Men with a body mass index (BMI) <18.5 or >30 kg/m2 and women with a BMI <18.5 or >40 kg/m2; prostate cancer, prostate enlargement, prostate surgery; polycystic ovaries; treatment for cancer, thyroid, testicular/ovarian or pituitary conditions in the past year; medication for epilepsy; women reporting both hysterectomy and HRT use; HRT; and hormonal contraception. All self-reported. See 'study population' section of methods for further details of exclusion criteria. | |
A) Men
B) WOMEN
B) Women
